# Pulmonary Tuberculosis in Children: A Forgotten Disease?

**DOI:** 10.3390/microorganisms11071722

**Published:** 2023-06-30

**Authors:** Umberto Pelosi, Roberta Pintus, Salvatore Savasta, Vassilios Fanos

**Affiliations:** 1 Pediatric Unit, Santa Barbara Hospital, 09016 Iglesias, Italy; 2 Neonatal Intensive Care Unit, Department of Surgical Sciences, University of Cagliari, AOU Cagliari, 09124 Cagliari, Italy; 3 Department of Pediatrics and Rare Diseases, Ospedale Microcitemico Antonio Cao, University of Cagliari, 09124 Cagliari, Italy

**Keywords:** children, pathogenesis, diagnosis, skin tests, Xpert MtB/Rif Ultra, therapy, prevention

## Abstract

Even today, tuberculosis in childhood is a disease that is often undiagnosed and undertreated. In the absence of therapy with antituberculosis drugs, children in the first years of life have a high degree of severe forms and mortality. In these children, symptoms are often not very specific and can easily be confused with other diseases of bacterial, viral or fungal etiology, making diagnosis more difficult. Nevertheless, the introduction of new diagnostic techniques has allowed a more rapid identification of the infection. Indeed, Interferon gamma release assay (IGRA) is preferred to the Mantoux, albeit with obvious limitations in children aged <2 years. While the Xpert Mtb/RIF Ultra test is recommended as an initial diagnostic investigation of the gastric aspirate and/or stools in children with signs and symptoms of pulmonary tuberculosis. The drugs used in the treatment of susceptible and resistant TB are the same as those used in adults but doses and combinations are different in the pediatric age. In children, brief therapy is preferable in both the latent infection and the active disease, as a significant reduction in side effects is obtained.

## 1. Introduction

To date, about 1.5 million children contract tuberculosis (TB) every year. They represent 1.1% of the entire affected population, of these in 2020, only 36.5% were notified to the competent authorities. TB is currently considered one of the 10 causes of death in childhood as stated in the WHO Global Tuberculosis Report. Furthermore, this document stated that, in 2020, TB was responsible for 16% of global deaths in children. The main cause of these deaths is above all the reduction, particularly in the most endemic countries, of access to health facilities and the adherence to therapeutic protocols. In Italy in 2020, 2287 cases of TB were recorded, 1.8% aged <5 years, 2.8% aged 5–14 and 15.1% aged 15–24 [1,2,3]. Currently, it is considered one of the 10 causes of death in childhood. In the course of the pandemic of SARS-CoV-2, in 2020, a sharp drop occurred in access to diagnosis and therapy services for children aged 5–15 years (63%) and a clear reduction in prophylaxis for tuberculosis infection in children < 5 years of age (72%). The reduction in access to hospital facilities has consequently led to a significant increase in new cases and deaths in the following years [4,5,6]. Furthermore, children are more vulnerable to tuberculosis infection because of their immunological immaturity characterized by an altered innate immunity in the lungs, due to a reduced recruitment of macrophages to the site of infection and a decreased production of IL-12, which is essential for the initial polarization of the Th1 lymphocytes, which, in turn, are important to maintain an efficient transcription of IFN-γ [7,8,9].

Moreover, children aged <5 years are more exposed to the infection and to the most severe forms of the disease, indeed about, in pediatric age, 80% of TB deaths in children occur mainly in this age group. Sometimes these children have silent forms of TB with mild non-specific symptoms and do not develop the disease. In these cases, there is a 10% risk of reactivation in adulthood. In addition, in children, the disease has some peculiarities that differentiate it from the adult forms [10,11,12]. These features are paucibacillary; a low incidence of cavitary forms; an increased frequency in evolution of the infection into the disease; a more rapid and severe course in children < 2 years; a high incidence of extrapulmonary forms; in 20–60% of cases it can be symptomatic; radiology is often of little significance; and diagnostic difficulties. Some of these are discussed in the following paragraphs.

## 2. Pathogenesis

*Mycobacterium tuberculosis* (MTB), an intracellular pathogen, is transmitted from a sick adult through exhaled air and the emission of droplets of infected secretions via sneezing and/or coughing. These droplets are particles measuring 1–5 microns that can transport 2–3 bacteria; the infection charge is around 10–150 bacteria; coughing, singing and talking for 5 min can produce 3000 droplets [13,14]. This microorganism has a cell wall that is rich in lipids, which confers some particular characteristics: acid resistance, slow growth and the possibility of surviving inside macrophages [15]. Moreover, a child is hardly able to infect another child. Indeed, in children they are the cavitary forms except in those with HIV or with genetic modifications in the immune response. The infection begins when the MTB enters the upper airways via inhalation where 90% is expelled by the mucociliary system and by the immune cells present in the lamina propria [16,17]. After bypassing the upper airway defense system, MTB enters the lungs usually in the middle lobe and lower lobes reaching the alveoli (<10%) where it encounters alveolar macrophages, dendritic cells and neutrophils, which binds to their surface [18,19,20]. 

**Figure 1:**
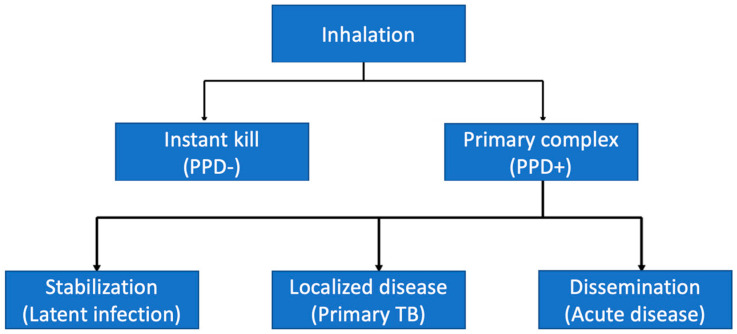
Chronological events after MTB inhalation.

However, this exposure leads to two events: the elimination of the germ or its persistence, and about 10% of infected people have a chance of developing the active disease [21,22]. These events are illustrated in Figure 1. 

An inadequate immunological response or an age < 2 years facilitate continuous mycobacterium replication and its progression to lung disease or its dissemination to other districts (Figure 1). On the other hand, children aged 5–10 years have a low risk of disease progression, while adolescents have a high risk of disease reactivation [23]. Table 1 illustrates the risk of progression of TB according to age (Table 1). 

Moreover, there are several factors that favor the transition from infection to disease and its severity, summarized in Table 2 [24]. 

From an immunological point of view, both innate and cell-mediated immunity are fundamental to contain the TB infection and their interplay contributes to the clinical presentations of TB [25,26]. The inhaled MTB, in immunocompetent subjects, binds to the surface of neutrophils and alveolar macrophages [27,28]. Neutrophil phagocyte MTBs kill them following the release of lysosomal enzymes and the production of reactive oxygen intermediates (ROI), human neutrophil peptides (HNPs), myeloperoxidase (MPO), neutrophil serine protease (NSP) and lactoferrin (LA) [29,30]. Macrophages recognize MTB by PPR3 membrane receptors which, when stimulated, via intracellular messengers activate transcription, leading to the production of proinflammatory cytokines and chemokines. In turn, this leads to an increased vascular permeability, causing the recruitment of other inflammatory cells, in particular dendritic cells. Thus, phagocytosis is activated through various mechanisms: (a) recognition of the mannose residues of the MTB by the receptors of the macrophage where the lipoarabinomannan present on the surface binds, (b) activation of the C3 proteins of the complement present in the alveolar space, which are recognized by the complement receptors present on the surface of macrophages [31,32,33].

Thus, MTB is phagocytosed inside the vacuoles and following the secretion of some cytokines and chemokines (IFN-γ, TNF-α, IL-1β, IL-6 and IL-23) is killed or limited in its growth [34]. Furthermore, most of the macrophages control the infection by rapidly activating some antimicrobial mechanisms: (a) fusion of phagosomes with lysosomes (formation of the phago-lysosomal complex), (b) autophagy, and (c) oxidative stress [33,34,35]. Nevertheless, bronchoalveolar cell responses featured in TB patients consist of lymphocytic alveolitis and immature macrophage alveolitis. This initial response involving innate immunity is sufficient to eliminate MBT. 

However, it tries to survive inside the host by inhibiting: (a) the fusion of phagosomes with lysosomes, (b) the maturation of macrophage phagocytosis by suppressing phagosome acidification and (c) the production in macrophages of ROS by catalase-peroxidase (Kat G), by reducing the action of oxidative stress [36,37,38,39]. Then, it is able to replicate inside the macrophages and to invade the pulmonary interstitium.

For their part, macrophages and dendritic cells, resident in the pulmonary interstitium, phagocytize MTB and migrate to the lymph nodes where they present MTB antigens to T cells that differentiate into CD4+ and CD8+ [40,41]. Then, the CD4 and CD8 cells return to the lungs thanks to the chemokines produced at the site of infection. Here, these activated T cells bind to the MHC/antigen complexes on the surface of the infected macrophages and produce a range of cytokines, including IFNγ, leading to the further activation of macrophages that kill MTBs that escaped the initial phagocytosis [39,42]. Moreover, TNF-α produced by macrophages, T cells, dendritic cells, fibroblasts and endothelial cells plays a key role in the formation of the granuloma at the site of the infection [43,44].

Indeed, the accumulation of lymphocytes, macrophages, dendritic cells and epithelioid cells at the site of the infection determines the formation of the granuloma within which the MTB is contained and where it can persist for several decades. In healthy close contacts of TB patients and during active pulmonary TB, the immune response is confined to the lung and characterized by an exuberant Th1 lymphocyte response, which is counteracted by local suppressive immune mechanisms, as suggested by the recent evidence [45]. Generally, a patient with a primary MBT infection is asymptomatic and undergoes clinical but not biological recovery, remaining infected with quiescent mycobacteria. This clinical quiescence often persists for the entire life of the individual. In a smaller percentage of cases, subjects with tuberculosis infection may immediately manifest the active disease (primary tuberculosis) or undergo a clinical reactivation of the infection during their life (secondary tuberculosis), due to the lack of development or maintenance of an effective immune response.

## 3. The Role of Microbiota

In the past, the scientific community believed that the lungs of healthy subjects were germ-free and that they could only be colonized in cases of lung pathology. This misconception resulted in the exclusion of the lung from the Microbiome Human Project [46]. Only starting in 2010, an increasing number of studies have shown, through new DNA and RNA investigation techniques, that the lungs of healthy subjects are not germ-free but are colonized by bacteria, viruses and fungi [47,48]. These new techniques lead to the identification of different species of bacteria: (1) at the phylum level: Firmicutes, Bacteroides and Proteobacteria, (2) at the genus level: *Veillonella*, *Prevotella*, Fusobacteria and Streptococcus, with the presence of small quantities of pathogens, such as *Haemophilus* and (3) fungi: *Aspergillus*, *Cladosporium*, *Penicillum* and *Eurotium* [48]. The lungs are colonized by different species in their different the anatomical region (bronchi, bronchioles and alveoli) thus, the constituents of the lung microbiota depends on several factors, some of which are particularly important, such as (1) microbial immigration (micro-aspiration, inhalation of microorganisms, direct mucosal dispersion), (2) microbial elimination (cough, mucociliary clearance, innate and adaptive immunity) and (3) regional growth conditions (nutritional availability, temperature, O2-tension, local microbial competition, concentration and activity of inflammatory cells) [49]. The equilibrium of these elements is essential for the composition of the lung microbiota in healthy subjects. The local factors in a healthy subject determines the adverse growth and a multiplication environment for the microorganisms. On the other hand, when the environment shifts, the reproduction of pathogens and the subsequent occurrence of pathologies, which can often become chronic, is favored. Moreover, the microbiota present in the human body produces metabolites capable of modulating the host’s immune response. Even microbial communities present at distal sites of the body play an important role in respiratory health. 

Furthermore, a theory has been advanced, namely that some essential risk factors for tuberculosis (HIV, malnutrition, diabetes, alcohol, smoking and pollution) interact with the host through the microbiota [50,51].

In addition, recent scientific evidence proves the contribution of the microbiota in TB pathogenesis, therapy response, clinical outcomes, and post-treatment outcomes [52]. This role is underpinned by complex multifactorial interactions between the pathogen, the commensal flora and the guest.

In particular, some peculiar aspects have been identified:

(1) Susceptibility to infection and progression to active TB is modified by intestinal Helicobacter co-infection;
(2) Airborne *Mycobacterium tuberculosis* infection modifies the intestinal microbiota;
(3) Anaerobes present in the lungs and coming from the oral cavity by aspiration produce metabolites that reduce lung immunity and predict progression;
(4) The increased susceptibility to reinfection of patients who have been previously treated for TB is probably due to the depletion of antigenic epitopes for T cells in the commensal intestinal flora (non-tuberculous mycobacteria);
(5) The prolonged antibiotic treatment necessary for tuberculosis has long-term detrimental effects on the microbiome.

Moreover, numerous evidences suggest that the gut microbiota modulates the host immune response against MTB and that this mechanism could constitute a potential therapeutic target [52].

Additionally, subjects affected by TB present a greater abundance of *Streptococcus*, *Gramulicatella* and *Pseudomonas.* While *Prevotella*, *Leptotrichia*, *Treponema*, *Catonella* and *Coprococcus* are less represented in subjects with TB than in healthy controls.

Moreover, subjects with recurrent TB show a reduced frequency of some genera, such as *Bulleidia* and *Atopobium* compared to subjects with new onset TB.

Finally, it has recently been hypothesized that the microbiome through the gut/lung axis plays an important role in granuloma formation, promoting the Th1 and Th17 response [52]. 

### Effects of Therapy on the Microbiota 

Several studies have demonstrated that the antibiotic administration is responsible for the alterations in both the intestinal and pulmonary microbiota [53]. The risk is inversely related to the age of exposure and to the amount and classes of antibiotics and determines important modifications in the immune response of the host against the pathogens, favoring the progression and severity of the disease [54]. As observed in other diseases, the administration of these drugs is responsible for the modifications in the intestinal and pulmonary microbiota [55]. While the effects on the intestinal microbiota are well documented, at present, there are few studies investigating the pulmonary dysbiosis [56]. This scarceness of information is closely correlated with the variety of pulmonary conditions that can arise in the course of lung injury (fibrosis, cavitations, broncho-stenosis, bronchiectasis, alteration of the parenchyma) in relation to age, diffusion and severity of the disease. The first particular evidence present in most of the investigations is that therapy with isoniazid, rifampicin, pyrazinamide and ethambutol (HRZE) has minimal effects on the diversity of the phyla of the intestinal microbiota but determines evident alterations on their distribution and quantity [57]. 

Animal studies and studies performed on humans with tuberculosis treated with HRZE show an increase in the genus of the phyla Bacteroides and Proteobacteria and a significant reduction in the phylum Firmicutes (*Clostridiales*) [58,59]. Another particular aspect of the alterations of the microbiota related to the therapy administration is the persistence of dysbiosis for 1 to 3 years after its suspension (chronic effects). Markers of persistent dysbiosis are the reduction in Bacteroides, Firmicutes (*Clostridiales*, *Ruminococcus*, *Faecalibacterium*) and an increase in Actinobacteria and Proteobacteria (*Escherichia*, *Salmonella*, *Yersinia*, *Helicobacter*) [60]. 

It has been speculated that the acute and chronic dysbiosis that occurs during therapy may be responsible for three possible conditions:

(1) Alteration of the host immune response that can influence the course and severity of the disease. Dysbiosis significantly reduces the immune response against MTB and leads to an increase in inflammation in the course of the disease. The reduction in Bacteroides and Firmicutes, in particular of *Ruminococcus* and *Coprococcus*, which regulate the expression of IL-1 and IFN-γ and of Bifidobacterium, which induces a reduction in the activity of Th17 cells, are responsible for these alterations [61]. Finally, another significant effect of dysbiosis is the decrease in the bactericidal effect of macrophages due to a decrease in their autophagy [62,63];
(2) Modification of the therapy response: there is no significant evidence in the literature to confirm the importance of dysbiosis on the efficacy of drugs, although it is hypothesized that the modifications affecting the intestinal mucosa and its barrier function may cause a reduced absorption and metabolism of the drugs themselves [64];
(3) An increased risk of reinfection: the persistence of dysbiosis, years after the suspension of therapy, seems to be responsible for an increased risk of reinfection [65]. It is commonly believed that the lower resistance of the host against MTB is related to an individual profile of the host microbiota, its profile before the disease or individually modified by therapy, which interacts with specific epitopes of the pathogen.

## 4. Vitamin D and Tuberculosis

Numerous studies have shown that vitamin D is capable, as occurs in other infectious processes, of modulating the immune response against MTB, in particular through the activation of No/ROS, which determines the inhibition of MTB growth in infected macrophages and LL-37, which modulates the production of proinflammatory cytokines. In detail, there is an increase in IL-10 and IL-12 and a decrease in TNF-α, with consequent reduction in the inflammatory state and tissue damage induced by MTB [14,66].

## 5. Diagnosis

Nowadays, diagnosis in children is often difficult, as the signs and symptoms, especially in the first years of life, can be confused with other pathologies.

The diagnosis is based on 1. Anamnesis, 2. Clinical criteria, 3. Skin Test 4. New diagnostic tests 5. Bacteriological tests and 6. Radiological investigations.

### 5.1. Anamnesis

The medical history of a contact with known cases of TB allows to suspect infection or disease. In children and in particular in those aged <2 years, the symptoms are often not very specific and can easily be confused with other diseases of bacterial, viral or fungal etiology, making the diagnosis more difficult [9,67]. Signs and symptoms that can guide the diagnosis are reported in Table 3.

The simultaneity of some symptoms, such as persistent cough, listlessness and weight loss, have a predictive value in 80% of children with pulmonary TB [11,68]. 

### 5.2. Clinical Criteria

**Figure 2:**
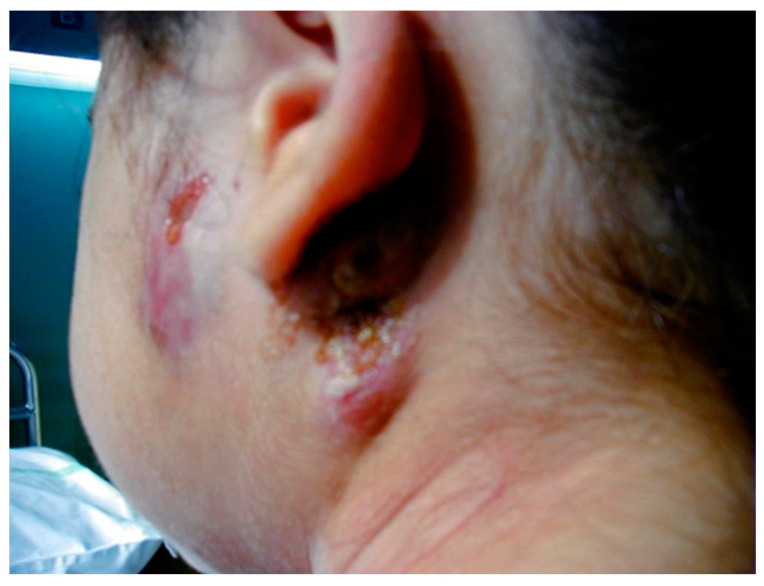
Scrofuloderma.

Symptoms vary according to the age of the child and the affected site. The pulmonary localization appears to be the most frequent (71.8%), while the extra-pulmonary localization is present in about 20–30% of cases and includes the localization of the infection in different districts: central nervous system (meninges, parenchyma and spinal cord) [69], lymph nodes (lymphadenitis present in 30–40% of cases in the latero-cervical, supraclavicular, axillary and mediastinal, abdominal) [70], skeleton (spondylitis, osteomyelitis and arthritis) [71,72], genitourinary system (present in 3% of cases, indicator of disease reactivation) [73], kidney (presence of pyuria in sterile urine) [74], gastrointestinal system [75] and skin (scrofuloderma shown in Figure 2) [76].

### 5.3. Skin Tests

The conventional skin test (Mantoux) is certainly the oldest test that has allowed both diagnostic and epidemiological screening [77,78]. The performing is simple and requires only minimal attention from the operator. Positivity appears 2–10 weeks after the beginning of the infection and is considered positive in relation to the size of the wheal. Positive Mantoux Definition is described in Table 4. 

Nevertheless, the tuberculin test has some disadvantages that can make diagnosis difficult. These disadvantages are shown in Table 5. 

The main problem of the intra-dermal reaction is that it does not allow to distinguish between infection and disease and between vaccinated and infected [79]. Nonetheless, three new tests have recently been developed for the diagnosis of MTB: (a) Diaskintest, (b) C-Tb skin test and (c) Ec-test. These tests are based on the presence of two MBT antigens: CFP-10 and ESAT-6. They have the advantage over the traditional skin test of differentiating between positives from infection and positives from vaccination [80,81].

### 5.4. New Diagnostic Tests

Interferon-gamma release assay (IGRA) tests have been widely used in the last decade [82]. These are based on the measurement of the IFN-y produced by T-lymphocytes, following stimulation with two highly specific MBT antigens: ESAT-6 and CFP-10, which are absent in *M.bovis* and the BCG [10,83]. Two second generation tests are currently on the market: the Quantiferon-TB Plus and the T-SPOT.TB, which differ from the previous ones in that they use new antigens which are able to stimulate both CD4 and CD8+ [84,85,86]. They have some advantages over the traditional skin test (summarized in Table 6). 

These tests also do not allow to distinguish the latent infection from the disease, such as the Mantoux test, and they are not recommended in children in the first years of life as they have a low sensitivity in this age group [87,88,89].

Recently, new diagnostic tests based on the rapid determination of MTB DNA in the sample (NAAT nucleic acid amplification test and Xpert Mtb/RIF Ultra test). While the LF-LAM test (Lateral Flow Urine Lipoarabinomannan) is based on the identification of the liposaccharide antigen LAM present on the cell wall of MTB [35]. 

Among these tests, the most recommended [6] are the Xpert Mtb/RIF Ultra and the LF-LAM. The first is recommended as an initial test in pediatric ages in cases of pulmonary tuberculosis. It can be performed on various samples: feces (97–100% specificity and 32–90% sensitivity) and gastric aspirate (94% specificity and sensitivity of 71.5%). The Xpert Mtb/RIF Ultra assay appears to be useful, in addition to diagnosis, for the identification in a single assay of resistance-associated mutations for rifampin, isoniazid, amikacin and capreomycin [90,91,92,93]. While the LF-LAM test (Lateral Flow Urine Li-poarabinomannan) is indicated as an additional test in HIV positive children, presenting signs and symptoms of pulmonary tuberculosis and in those who are HIV positive without symptoms with a CD4 cell count < 100/mm^3^ [6,8].

### 5.5. Bacteriological Tests

The diagnostic gold standards are certainly the MTB culture and the polymerase chain reaction (PCR) but the difficulty, for several reasons, in obtaining appropriate examination samples in children represents the major obstacle to this investigation, since the disease is often paucibacillary and children, unlike adults, hardly produce sputum and the bronchoscopy has considerable limitations [94,95,96]. Therefore, non- or minimally invasive tests on sputum (the emission is favored by performing an aerosol with hypertonic salic solution), on gastric aspirate, nasopharyngeal secretion, on urine and recent feces, are recommended [97]. Recent studies have shown that the examination of multiple samples increases the diagnostic sensitivity [98]. In children, due to low bacterial load, the MTB culture has a sensitivity only of 7–40% and requires at least 2–4 weeks for growth, which limits the use of this assay [27]. It is strongly recommended to carry out the culture by combining solid and liquid media [99].

### 5.6. Radiological Investigations

The first line investigations are radiography and ultrasound of the affected site but both may have limitations and low sensitivity, especially ultrasound since it is a highly operator-dependent imaging modality [100,101]. Especially in the first years of life, the radiological picture can be confused with other diseases and the thymus silhouette can complicate the evaluation of the mediastinum when enlarged lymph nodes are present. It is always advisable to perform an anteroposterior and lateral chest X-ray [102]. A Computed Tomography scan and Magnetic Resonance Imaging may often be needed [103,104]. Radiographic changes suggestive of TB include [105]: enlargement of perihilar or paratracheal lymph nodes, alveolar thickening, pulmonary miliary, cavitations (more frequent in adolescents) and pleural or pericardial effusions. In the case of pulmonary tuberculosis, chest X-rays should be repeated 1–2 months after the beginning of therapy. In some cases, a worsening can be observed at the first X-ray control, despite the presence of an evident improvement in the clinical picture. In these cases, it is advisable to repeat the X-ray after 1–2 months and at the end of therapy [106].

## 6. Therapy

The main objectives of therapy are to prevent the progression from infection to disease and to cure the active form, in view of the fact that the risk of the onset of serious forms remains high even 2 years after infection and is particularly high in children aged <2 years. The drugs used in the treatment of susceptible and resistant TB are the same used in adults, they vary in pediatric age both in doses and combinations. The drugs classification is shown in Table 7.

Compared to adults, children have fewer side effects related to therapy. Indeed, the risk of hepatotoxicity is greater only in obese children (hepatic steatosis) and with the concomitant administration of anticonvulsant drugs. For these children, it is advisable to determine liver function before starting treatment and 1–2 months after the beginning. Isoniazid (INH), rifampicin (RIF), pyrazinamide (PZA), ethambutol (EMB), rifabutin and rifapentine are the drugs mostly used both in the therapy of latent infection and in the active disease. Recently, it has been recommended to increase the dosage of some of these drugs: IH 10–15 mg/Kg, RIF 10–20 mg/Kg, PZA 30–40 mg/Kg and EMB 15–25 mg/Kg [6].

### 6.1. Latent Infection

The latent infection is symptomatically silent and can be suspected if there has been contact with an adult with the active disease.

Various therapeutic regimens are currently proposed [107,108]:

(1) Isoniazid + rifapentine:
  - Age: 2–11 years;
  Isoniazid 25 mg/Kg (max 900 mg) +
  Rifapentine 5 mg/kg 1 dose/week per 3 months (12 doses)
  - Age ≥ 12 years;
  Isoniazid 15 mg/Kg (max 900 mg): 1 dose/week per 3 months (12 doses)
  Rifapentine weight
  Kg:
  10–14: 300 mg
  14.1–25: 450 mg
  25.1–32: 600 mg
  32.1–49.9: 750 mg
  ≥50: 800 mg

This regimen is not recommended in children < 2 years of age and has the same preventative effects as isoniazid treatment for 6 or 9 months. It is preferred over isoniazid monotherapy in children with a high risk of hepatotoxicity.

(2) Rifampicin 15–20 mg/Kg/die (max 800 mg/die) per 4 months. It has similar results to 6 or 9 months of isoniazid [109];
(3) Isoniazid 25 mg/Kg/die + Rifampicin 15–20 mg/Kg/die per 3 months. It is recommended in children aged <5 years since the benefits of using this combination significantly outweigh the risks;
(4) Isoniazid 10–15 mg/Kg/die (max 900 mg die) for 6 months;
  Isoniazid 20–30 mg/Kg/die (max 300 mg/die) 2 times/week for 6 months;
(5) Isoniazid 10–15 mg/Kg/die (max 300 mg/die) for 9 months;
(6) Isoniazid 20–30 mg/Kg/die (max 900 mg/die) 2 times/week per 9 months.

Isoniazid prophylaxis has greater hepatotoxic effects than rifampicin and rifapentine therapy.

During therapy, it is advisable to perform a follow-up after the first month in order to evaluate:

- The presence of symptoms or signs indicative of progression towards the disease;
- The presence of adverse effects to the administered drugs and it is advisable to perform a blood count and the dosage of transaminases.

### 6.2. Active Disease

The drugs used in the therapy of the active disease are:

- Isoniazid (H) 10 mg/Kg/die (max 300 mg/die);
- Rifampicin (R) 15 mg/Kg/die (max 600 mg/die);
- Pyrazinamid (Z) 35 mg/Kg/die;
- Ethambutol (E) 20 mg/Kg/die.

Therapy schemes [1]:

(1) Children with suspected or confirmed pulmonary tuberculosis or peripheral lymphadenitis living in environments with low incidence of HIV and isoniazid resistance:
  HRZ for 2 months followed by HR for 4 months.
(2) Children with suspected or confirmed pulmonary tuberculosis or peripheral lymphadenitis or children with disseminated lung disease living in environments with high incidence of HIV and isoniazid resistance: HRZE for 2 months followed by HR per 4 months;
(3) Children with suspected or confirmed TB meningitis or with suspected or confirmed osteoarticular TB: HRZE for 2 months followed by HR for 10 months;
(4) A short course of therapy (2 months with HRZ followed by 2 months with HR) has recently been proposed in children aged 3 months to 15 years with non-severe TB [110].

Steroid therapy (dexamethasone and prednisolone) is indicated in meningitis and TB pericarditis. The administration of vitamin B6 is recommended both in the treatment of latent infection and of the active disease.

### 6.3. Treatment of Drug Resistant TB

Resistance to antituberculosis drugs is rare in children, with an estimated 25,000–32,000 children worldwide each year developing drug resistance. Several studies conducted in Europe have shown that the increase in immigration has coincided with an increase in resistant TB cases. The diagnosis of these forms is particularly difficult in children because it is not always possible to isolate the bacteriological material (paucibacillary) to identify resistance to various drugs. According to WHO [111], four definitions of drug resistance are distinguished: 1. Resistance to a drug, 2. Mono-resistance to isoniazid (9%) (susceptibility to rifampicin), 3. Resistance to rifampicin (1%) [112] and, 4. Multidrug-resistance (at least isoniazid and rifampicin) [113,114]. These children with drug resistant bacteria are treated with the same antibiotic combinations used in adults (see Table 7). The WHO has recently authorized the administration of two new drugs: bedaquiline and delamanid [115]. Bedaquiline is a bactericidal agent that interferes with the ATP synthesis of the MTB. It can be used in children of all ages. The therapy with this drug consists of a dose of 6 mg/Kg once a day for 2 weeks followed by 3–4 mg/Kg three times a week (max dose 200–400 mg) [116]. It is recommended for 9–12 months in multidrug-resistant and rifampin-resistant forms. Its long half-life allows intermittent administration when combined with other drugs used in resistant forms. Delamanid is a bactericidal agent of the nitroimidazole class which, through nitro-reductases, reduces the synthesis of neicholic acid and inhibits MB replication. The recommended dose is 3–4 mg/kg in one or two doses a day in children aged <3 years, 25 mg twice a day in children aged 3–5 years, 50 mg twice a day in children aged 6–11 years and 100 mg twice daily in ages 12–17 years [117]. It is recommended for 6 months and can be used for a maximum of <18 months. During treatment, ECG and electrolyte monitoring and neuropsychiatric monitoring are recommended (risk of hallucinations).

## 7. Vaccine

Currently, BCG (Bacillus Calmette–Guerin) is still the only available vaccine in the world [118]. It is particularly active in children in preventing the onset of the most serious forms (meningitis and miliary) in 50% of cases and pulmonary disease in 80% of cases, while it is unable to prevent the reactivation of the latent pulmonary infection. It can cause disseminated forms when administered to immunodeficient and HIV positive children. As BCG’s protection against lung infection decreases with age, there is the need for new vaccines. Today, several new vaccines derived from killed mycobacteria, live vaccines attenuated by genetic modifications and recombinant vaccines with viral vectors are being tested and must complete the trial cycle to be approved [119].

## 8. Conclusions

TB is still a dangerous disease in childhood, as children represent the population at greatest risk of morbidity and mortality. Furthermore, the COVID-19 pandemic has highlighted how the decrease in access to health facilities has led to a significant delay in diagnosis and treatment, contributing to an increase in deaths and new cases in the following years [120]. For what concerns the diagnosis in children, it is not always simple, especially in the first years of life, even if the introduction of new diagnostic techniques has allowed a more rapid identification of the infection. The use of the IGRA is preferred to the Mantoux, albeit with obvious limitations in children aged <2 years. While the Xpert Mtb/RIF Ultra test is recommended as an initial diagnostic test of gastric aspirate and/or stools in children with signs and symptoms of pulmonary tuberculosis. At present it is commonly believed that there is an interrelationship between the microbiota, the tuberculosis disease and therapy. MBT infection is linked to a peculiar and complex immunological response, which is microbiota dependent, the profound modifications mainly affecting the intestinal microbiota, are responsible for a lower resistance of the subject against *M. tuberculosis.* From the imaging point of view, the chest X-ray is now an important examination in children with pulmonary tuberculosis with symptoms but negative tests. Moreover, it is important to perform the lateral view in children < 5 years of age as well.

In addition, children represent the most suitable population for brief therapy both in the latent infection and in the active disease since a significant reduction in the side effects is obtained. 

Finally, in recent years, a greater increase in drug-resistant TB cases has been observed, partly related to immigration from high-incidence countries. Resistance to antituberculosis drugs is rare in children, with an estimated 25,000–32,000 children worldwide each year developing drug resistance. It is believed that new studies are needed to better define the therapeutic approach in drug resistant forms, especially in the age < 5 years.

## Figures and Tables

**Table 1:** Risk of progression of TB according to age.

Age	Risk (%)
<12 months	40–40
1–2 years	25
School age	5–10
Adolescents	10–15
Adults	5–10

**Table 2:** Risk factors for tuberculosis in children after the infection.

Risk Factors for the Transition from Infection to Disease TB in Children
Presence of chronic cough Extrapulmonary tuberculosis Poor use of health facilities First non-specialist physician examination, such as family pediatrician with no experience with TB in children Immigration history Low socio-cultural level

**Table 3:** Signs and symptoms of tuberculosis in children.

Signs and Symptoms of Tuberculosis in Children
persistent cough > 4 weeks fever (often low-grade and intermittent) night sweats weight loss and anorexia general malaise and asthenia hemoptysis chest pain (if there are subpleural lesions) respiratory distress pneumonia resistant to antibiotic therapy

**Table 4:** Positive Mantoux definition in children.

Positive Mantoux Definition in Children	
Reaction ≥ 5 mm	Children in close contact with a person with a known or suspected tuberculosis infection Children with suspected tuberculosis disease Chest X-ray consistent with disease Clinical signs of tuberculosis Children receiving immunosuppressive therapy or with congenital or acquired immunodeficiency
Reaction ≥ 10 mm	Children at increased risk of disseminated form: age < 4 years with risky clinical conditions (lymphomas, diabetes, malnutrition, etc.) Children with increased risk of environmental exposure: Born in, or with parents coming from, highly endemic areas Frequently exposed to adults with HIV, drug addicts, inmates Travel and exposure to regions of the world with high prevalence
Reaction ≥ 15 mm	Children ≥ 4 years without risk factors

**Table 5:** Factors that may make the evaluation of Mantoux difficult.

Factor That Can Affect the Mantoux Evaluation	
Positive	BCG vaccination Non-tuberculous mycobacteria (NTM)
Negative	measles vaccination temporary anergy (2–12 weeks after infection), previous administration of Corticosteroids or immunosuppressants

**Table 6:** Comparison between Mantoux and IGRA.

	Mantoux	IGRA
Recommended age	All	>2 years
Cross reactivity with BCG	YES	NO
Distinction between infection and disease	NO	NO
Cross reactrivity with non tubercular Mb	YES	Not Much
Operator	Dependent	Not dependent
Cost	Low	High

**Table 7:** Drugs classification.

Group A	Group B	Group C
Levofloxacin/moxifloxacin Bedaquiline	Clofazimines Cycloserine/terizidone	Ethambutol, Amikacin/streptomycin Delamanid, Ethionamide/prothionamide Pyrazinamide, P-aminosalicylic acid, Imipenem, Meropenem, Amoxicillyn + clavulanic acid

## Data Availability

Not applicable.
